# Impact of an Educational Intervention on Public Perception of Health Problems in Brachycephalic Dogs

**DOI:** 10.1002/vms3.70399

**Published:** 2025-05-19

**Authors:** Gareth Smith, Alison P. Wills

**Affiliations:** ^1^ Department of Animal and Agriculture Hartpury University Gloucestershire UK

**Keywords:** dogs, education, welfare

## Abstract

**Background:**

The popularity of brachycephalic dog breeds has increased over the last decade despite their numerous health issues. The continued popularity of these breeds raises questions about public understanding and perception of their health problems.

**Objectives:**

This study aimed to determine the impact of a minimalist educational intervention on public awareness of the health of brachycephalic breeds and attitudes to breeding practices.

**Methods:**

Data were collected via an online questionnaire that assessed awareness of health issues and attitudes to breeding brachycephalic breeds before and after an educational intervention. The intervention consisted of a factsheet that survey participants were required to read. Kruskal–Wallis tests were used to test for an effect of dog ownership status (dog owner, owner of a brachycephalic breed, or not a dog owner) on perceptions, and Wilcoxon signed‐rank tests were used to test for the effect of the intervention on the perceptions of each individual group.

**Results:**

There were 141 survey responses obtained from owners of brachycephalic breeds (*n* = 41), owners of other breeds (*n* = 88), and non‐dog owners (*n* = 12). The intervention significantly improved the awareness of breed‐related health issues in owners of non‐brachycephalic breeds (*p* < 0.001) and non‐dog owners (*p* = 0.016) but not in owners of brachycephalic dogs (*p* = 0.581). Owners of non‐brachycephalic breeds significantly reduced their support for breeding brachycephalic dogs following the intervention (*p* < 0.001), but there was no significant effect on participants who did not own a dog (*p* = 0.059) or who owned a brachycephalic breed (*p* = 0.359).

**Conclusions:**

This study has demonstrated that education can change public perception of breed‐specific health issues and attitudes to continued breeding of brachycephalic dogs. However, attitudes of owners of these breeds appear to be less easily influenced than people who do not already own these dogs. Therefore, further work is needed to understand the motivations for breed ownership and the continued popularity of brachycephalic dogs.

## Graphical abstract

An educational intervention did not affect awareness of health issues in owners of brachycephalic dogs but increased awareness in owners of non‐brachycephalic breeds and non‐dog owners. Support for breeding did not change in owners of brachycephalic breeds or in non‐dog owners but decreased in owners of other breeds.

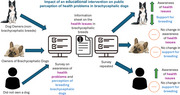

## 1 Introduction

The worldwide popularity of brachycephalic breeds has increased over the last decade including in the United Kingdom and the United States (Steinert et al. 2019; American kennel club 2020; Bognár and Kubinyi 2023). This rise has occurred despite the various health issues commonly associated with these breeds (O'Neill et al. 2023; Maclennan and Smith 2019). The growing ownership of brachycephalic dogs has led numerous researchers and organisations to express concerns about the health and welfare implications of the physical characteristics of these breeds (Rooney et al. 2009; Felska‐Błaszczyk and Seremak 2021; Paul, Packer et al. 2023). There is a growing body of evidence suggesting that these brachycephalic breeds are highly predisposed to many disorders that are intrinsically linked to their characteristic physical traits (Ekenstedt et al. 2020; O'Neill et al. 2020).

Brachycephalic breeds possess distinct phenotypic traits that lead to significant morphological alterations in a substantial proportion of dogs, making them easily recognisable (Bannasch et al. 2010). Brachycephalic dogs are characterised by a severe shortening of the muzzle and a less severe shortening and widening of the skull (Bannasch et al. 2010; Downing and Gibson 2018; Geiger et al. 2021). There is no current definitive list of brachycephalic breeds due to the differences in how brachycephaly is defined and measured (skull width‐to‐length ratio, craniofacial angle, craniofacial ratio, etc.) (Ekenstedt et al. 2020). The French Bulldog, Bulldog, Pug, Boston Terrier, and Boxer are popular breeds of brachycephalic dogs (Ekenstedt et al. 2020; Geiger et al. 2021). Although these breeds are often defined by cranial anatomy, they can often share other physical predispositions. A high prevalence of skin folds is seen in breeds such as French Bulldogs, Bulldogs, and Pugs (O'Neill et al. 2020, 2022). Additionally, a ‘screw tail’ is more prevalent in these breeds (Moissonnier et al. 2011; Schlensker and Distl 2013).

Health issues in brachycephalic dog breeds present a significant challenge, as there is increasing evidence that their exaggerated body morphology is directly associated with these issues (Ekenstedt et al. 2020; O'Neill et al. 2020). These conditions include a broad array of concerns, including respiratory disease, dermatological problems, eye disease, dystocia, spinal disease, heatstroke, and pneumonia (Hobi et al. 2023; Ryan et al. 2017; O'Neill et al. 2015, 2017; Packer et al. 2015). Moreover, research indicates that brachycephalic breeds typically have a lifespan that is approximately 3 years shorter than that of mesaticephalic and dolichocephalic breeds of comparable body size (Liu et al. 2017; O'Neill et al. 2015).

Despite the widely recognised health issues faced by brachycephalic breeds, their continued popularity raises questions about the level of public understanding and perception of these health concerns (O'Neill et al. 2020). The term brachycephalic paradox is often used to describe this phenomenon where the ownership of these breeds has increased alongside the advancement in information regarding their health issues (Packer et al. 2019; Bognár and Kubinyi 2023). Multiple studies have attributed the current high demand for brachycephalic dogs to both the attractive ‘cute’ physical facial features and their desirable behaviour (e.g. ‘affectionate temperament’ and ‘overall good companion breed’) (McGreevy et al. 2013; Packer et al. 2017; Paul, Coombe et al. 2023). Furthermore, the appearance of brachycephalic breeds is reported to be a more influential factor when acquiring a dog than the dog's health and function (Packer et al. 2017; Maclennan and Smith 2019). Additionally, in a study by Maclennan and Smith (2019) that surveyed the owners of purebred brachycephalic dogs, 86.1% reported they did not seek advice from a veterinary professional before acquiring their dog, suggesting a potential lack of awareness of the health issues they may face. Packer et al. (2012) identified that more than half of owners with brachycephalic dogs showing symptoms of brachycephalic obstructive airway syndrome (BOAS) failed to acknowledge their pets were exhibiting breathing issues, often justifying the signs as ‘typical for the breed’. Additionally, Packer et al. (2019) found that only 6.8% of owners of the three most commonly registered brachycephalic breeds with the Kennel Club believed their dog to be ‘less’ or ‘much less’ healthy than the breed average. It is possible that this is due to a level of cognitive dissonance amongst owners of brachycephalic breeds (Packer et al. 2019; Pound et al. 2024).

There is conflicting information regarding the effectiveness of infographics and information interventions in changing public perception of the health issues innate to brachycephalic breeds. Studies by Bognár and Kubinyi (2023) and Steinert et al. (2019) concluded that education regarding health problems alone is not sufficient to discourage people from acquiring these dogs or reduce their popularity. However, to the best of the authors’ knowledge, only one study has directly analysed the effectiveness of an educational intervention, and this study focused solely on a single health issue (BOAS) (Kenny et al. 2022). This study found that 53% of all participants and 29.3% of owners of brachycephalic breeds experienced a change in their view of these breeds. Furthermore, the majority of participants (99.5%) believed that prospective owners should be better informed about BOAS. Therefore, the aim of the present study was to asses public perception of health issues in brachycephalic dogs, both before and after an educational intervention focussed on four common pathologies seen in these breeds.

## 2 Materials and Methods

A quantitative cross‐sectional study in the form of a questionnaire was used to investigate the effects of an information intervention on the public perception of brachycephalic dog breeds. Approval for this study was granted by the Hartpury University Ethics Committee (ETHICS2023‐284‐LR).

### 2.1 Recruitment Strategy

A mix of convenience sampling and snowball sampling were used to disseminate the questionnaire. The survey was available between 3 February 2024 and 26 February 2024. Recruitment was conducted via social media platforms, targeting a diverse audience through Facebook and LinkedIn, including breed‐specific groups, dog welfare communities, and special interest groups that were not related to animals. The objective was to ensure a broad range of participants with varying levels of awareness and opinions about brachycephalic dogs, in line with previous work (Kenny et al. 2022). Additionally, the questionnaire was distributed via the author's and colleagues' personal social media platforms, which aimed to broaden the diversity of respondents and mitigate potential sampling bias. Inclusion criteria required individuals to be over the age of 18 and resident in the United Kingdom which was clearly stated in the informed consent paragraph that was provided to participants prior to commencing the survey. Participants were also informed that they could withdraw from the study during completion of the questionnaire by exiting the survey. Due to the data being anonymous, withdrawal after survey submission was not possible.

### 2.2 Questionnaire Design

The study was hosted on Jisc Online Surveys. The survey aimed to evaluate the baseline understanding of brachycephalic dog health issues amongst the sample population and the effect of an educational intervention. The questionnaire defined brachycephalic dogs as short‐faced breeds such as the French Bulldog, Pug, English Bulldog, Boxer, Shih Tzu, and Boston Terrier. The questionnaire contained one demographic question asking respondents to select their age group. There were eight further multiple‐choice questions covering topics such as dog ownership, familiarity with the term brachycephalic, whether the information sheet was easy to understand, and opinions on public awareness of brachycephalic dog health issues. Participants were asked whether they had previously or currently owned a dog, and if so, whether it was a brachycephalic breed. The survey contained five Likert scale questions which asked respondents to rate their understanding of health issues faced by brachycephalic dogs, their support for the continued breeding of these dogs, and their likelihood of sharing this information with others. The Likert scale questions assessed the understanding of health issues commonly faced by brachycephalic dog breeds, ranging from one (no understanding) to five (comprehensive understanding), with intermediate options of two (some understanding), three (good understanding), and four (very good understanding). The degree of support or opposition to continued breeding ranged from one (strongly oppose) to five (strongly support), with options of two (slightly oppose), three (neither oppose nor support), and four (slightly support). The likelihood of sharing or discussing the information ranged from one (very unlikely) to five (highly likely), with intermediate options of two (unlikely), three (neither likely nor unlikely), and four (likely). The Likert scale questions on understanding of health issues and support for breeding were presented to respondents in an identical fashion before and after they had been shown and requested to read a single page information sheet. The survey also contained one optional qualitative question giving the participants the ability to provide any additional comments or thoughts on the topic.

### 2.3 Information Intervention

The intervention contained a one‐page information sheet designed to present information on the conformation and health challenges of brachycephalic breeds. It contained a brief history of breeding and a short explanation of the outcome of this specific breeding. The sheet also contained information on four common health concerns (respiratory, dermatological, ocular, and spinal issues). These were selected based on existing literature highlighting the significance and prevalence of these conditions in brachycephalic breeds (Costa et al. 2021; Kenny et al. 2022; Hobi et al. 2023; Sebbag and Sanchez 2023). Each section of the information sheet included a simple description of the pathologies and their management strategies, without incorporating images. To maximise the understanding of both the questionnaire and information intervention, language was simplified with complex scientific terms avoided.

### 2.4 Data Analysis

Data analysis was performed using the statistical software SPSS (version 29.0). Due to the ordinal nature of Likert scale responses, non‐parametric tests were utilised for statistical analysis. The Kruskal–Wallis test was applied to assess the effect of dog ownership status on perceptions of brachycephalic dogs before the educational intervention between three groups; dog owners, non‐dog owners, and brachycephalic breed owners. Where significance was found in the main test, Dunn's post hoc test with a Bonferroni correction applied was used for pairwise comparisons (Armstrong 2014). The Wilcoxon signed‐rank test was used to compare pre‐ and post‐intervention responses, allowing for an evaluation of the educational intervention's impact on participants' perceptions. The criterion of significance for all analyses was *p* < 0.05. The statistical software R version 4.2.1 was used for the creation of figures (packages: ggplot2, reshape and forcats) (R Core Team, 2023).

## 3 Results

A total of 141 respondents participated in the study. Participants were a range of ages, 12.77% (*n* = 18) were 18–24, 21.28% (*n* = 30) were 25–34, 17.02% (*n* = 24) were 35–44, 20.57% (*n* = 29) were 45–54, 15.60% (*n* = 22) were 55–64, and 12.77% (*n* = 18) were 65 or older. The majority of respondents 91.49% (*n* = 129) had previously owned a dog, while 8.51% (*n* = 12) had not. Amongst those who had owned a dog, 29.08% (*n* = 41) had owned a brachycephalic breed. Additionally, 84.00% (*n* = 119) of participants were familiar with the term ‘brachycephalic’. Of the respondents who had never owned a brachycephalic breed, 11.00% (*n* = 10) would consider owning one.

### 3.1 The Effect of Dog Ownership on Understanding of Health Issues Pre‐Intervention

**FIGURE 1:**
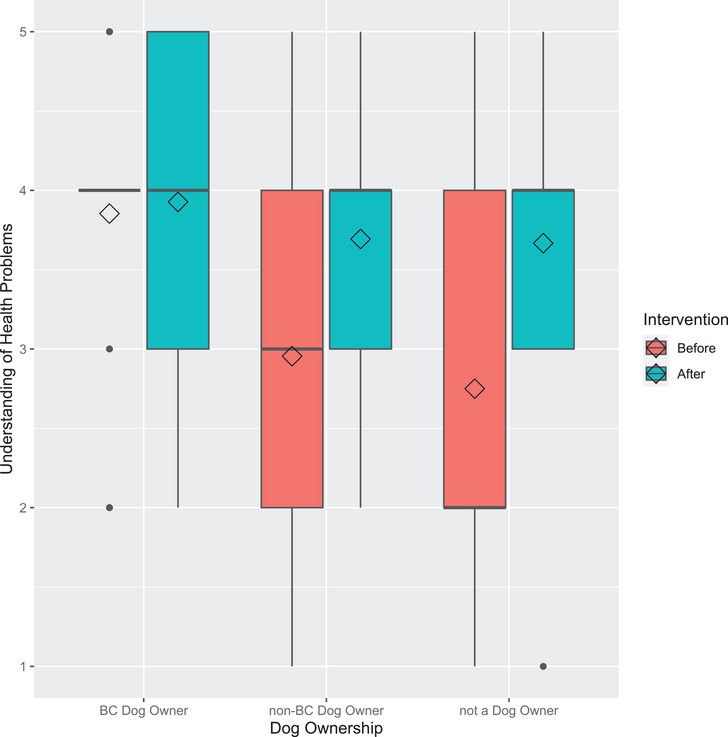
Understanding of health issues of brachycephalic dogs before and after the educational intervention. Participants self‐reported their understanding on a five‐point Likert scale from no understanding (1) to comprehensive understanding (5). Diamonds represent the mean. BC, brachycephalic.

Prior to the educational intervention, there was a significant main effect of dog ownership (dog owner, not a dog owner or brachycephalic dog owner) on self‐rated understanding of the health issues affecting brachycephalic breeds (*H*(2) = 20.693, *p* < 0.001). Participants who did not own a dog (2.75 ± 1.42; *p* = 0.008) and owners of other dog breeds (2.95 ± 1.06; *p* < 0.001) had significantly less understanding of health issues than brachycephalic breed owners (3.85 ± 0.91). There was no significant difference in understanding between participants who did not own a dog and those who owned a non‐brachycephalic breed (*p* = 1.000; Figure 1).

### 3.2 The Effect of the Educational Intervention on Understanding of Health Issues

Dog owners' understanding improved significantly from 2.95 ± 1.06 to 3.69 ± 0.84 after viewing the information sheet (*p* < 0.001). Non‐dog owners' understanding significantly increased from 2.75 ± 1.42 to 3.67 ± 1.07 after the infographic (*p* = 0.016). However, there was not a significant change in understanding before 3.85 ± 0.910 and after 3.93 ± 0.81 viewing the information sheet (*p* = 0.581) for owners of brachycephalic dogs (Figure 1).

### 3.3 The Effect of Dog Ownership on Understanding of Health Issues Post‐Intervention

After the educational intervention, there was no significant main effect of dog ownership (dog owner, not a dog owner, or owner of a brachycephalic breed) on self‐rated understanding of the health issues affecting brachycephalic breeds (*H*(2) = 1.883, *p* = 0.390).

### 3.4 The Effect of Dog Ownership on Support for Breeding Brachycephalic Dogs Pre‐Intervention

Prior to the educational intervention, there was a significant main effect of dog ownership (dog owner, not a dog owner, or owner of a brachycephalic breed) on support for breeding brachycephalic breeds (*H*(2) = 27.659, *p* < 0.001).

**FIGURE 2:**
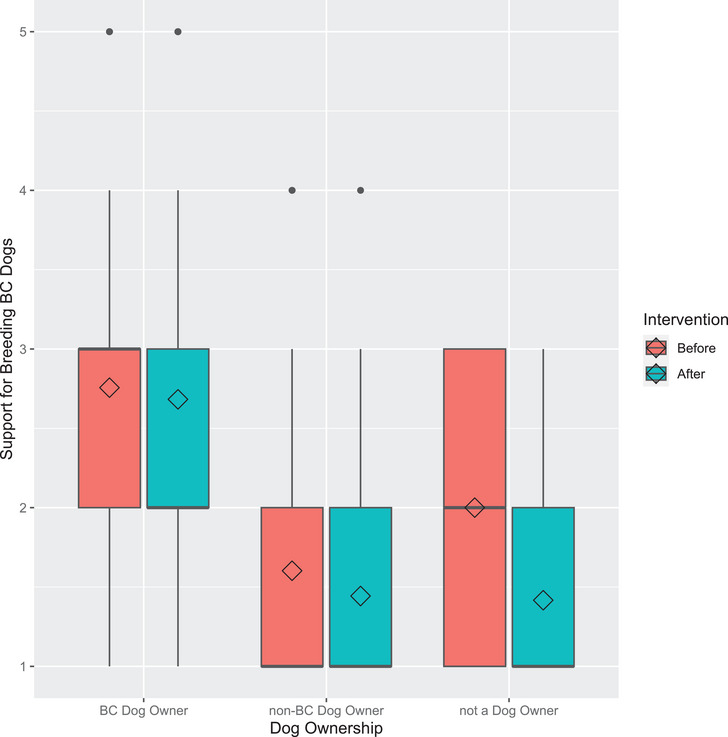
Support for breeding brachycephalic dogs before and after an educational intervention. Participants rated their support on a five‐point Likert scale from strongly oppose (1) to strongly support (5). Diamonds represent the mean. BC, brachycephalic.

There was no significant difference in support between dog owners (1.60 ± 0.78) and non‐dog owners (2.00 ± 0.85; *p* = 0.390). However, owners of brachycephalic breeds (2.76 ± 1.28; *p* < 0.001) were significantly more supportive of breeding than owners of other breeds (1.60 ± 0.78). There was no significant difference between non‐dog owners (2.00 ± 0.85) and owners of brachycephalic breeds (2.76 ± 1.28; *p* = 0.331) in their support for breeding brachycephalic breeds (Figure 2).

### 3.5 The Effect of the Educational Intervention on Support for Breeding Brachycephalic Dogs

Owners of non‐brachycephalic breeds’ level of support for breeding significantly reduced from 1.60 ± 0.78 to 1.44 ± 0.72 after viewing the infographic (*Z* = −3.300, *p* < 0.001). Non‐dog owners' level of support did not significantly change from before (2.00 ± 0.85) to after viewing the infographic (1.42 ± 0.66; Z = −1.890, *p* = 0.059). Owners of brachycephalic breeds showed no significant change in opinion before the infographic (2.76 ± 1.28) to after the infographic (2.68 ± 1.36; *Z* = −0.918, *p* = 0.359).

### 3.6 The Effect of Dog Ownership on Support for Breeding Post Intervention

After the intervention, there was a significant main effect of dog ownership (dog owner, not a dog owner, or owner of a brachycephalic breed) on support for breeding brachycephalic breeds (*H*(2) = 32.413, *p* < 0.001). Owners of brachycephalic breeds had greater support for breeding (2.68 ± 1.36) than owners of other breeds (1.44 ± 0.72; *p* < 0.001) and non‐dog owners (1.42 ± 0.67; *p* = 0.004). There was no significant difference between owners of non‐brachycephalic breeds (1.44 ± 0.72) and non‐dog owners (1.42 ± 0.67; *p* = 1.000).

### 3.7 The Impact of the Educational Intervention

Of the participants, 98.00% (*n* = 138) found the information provided easy to understand. About 99.00% (*n* = 139) understood the term brachycephalic after reading the information sheet. The majority of respondents (99.00%, *n* = 139) believed the public should be more informed about health issues of brachycephalic dogs, and 99.00% (*n* = 139) thought that potential owners should be made aware of these health issues before acquiring a brachycephalic dog. For the question ‘How likely are you to share or discuss the information provided with friends or family?’, *n* = 36 respondents (25.53%) indicated they were ‘Very Likely’ to share the information, *n* = 57 respondents (40.43%) indicated ‘Likely’, *n* = 36 respondents (25.53%) indicated ‘Neither Likely Nor Unlikely’, *n* = 7 respondents (4.96%) indicated ‘Unlikely’, and *n* = 5 respondents (3.55%) indicated ‘Very Unlikely’.

## 4 Discussion

The present study assessed the efficacy of a minimalistic information intervention that contained a brief description of four common health issues found in brachycephalic breeds. Before reading the information sheet, 84% of the sample population were familiar with the term brachycephalic, suggesting awareness of the classification of these breeds. Interestingly, the figure increased to 99% understanding of the term after the intervention. Consistent with previous work, 99% of participants believed the general public should be more informed about the health issues facing brachycephalic breeds (Kenny et al. 2022). The majority of participants (98%) found the information provided easy to understand which is reassuring as Lee and Lee (2021) suggested a simplistic and easy‐to‐understand design could have a crucial impact on the audience's attention and ability to retain information.

Prior to reading the information sheet, there was no statistically significant difference in understanding between people who did not own a dog and those who owned non‐brachycephalic breeds. This suggests that even though dog owners may have experience with their own breeds and any health problems associated with them, this does necessarily mean they are informed on breeds that they do not own. However, knowledge differed significantly between non‐dog owners and owners of brachycephalic breeds, as well as between owners of other breeds and owners of brachycephalic breeds, with the latter group showing higher knowledge. This is likely due to the owners of brachycephalic breeds having experience with their own dogs and their increased likelihood to be in communities that discuss these issues when compared to people who do not own brachycephalic breeds (Phillips et al. 2024). Another reason for the potential differences in knowledge arises if owners do not perceive health issues as a problem (Packer et al. 2012). Multiple studies have reported that owners and breeders of brachycephalic dogs often consider these health issues normal for the breed (Packer et al. 2012; Felska‐Błaszczyk and Seremak 2021). Lack of appreciation of the significance of health issues is a concern considering the high prevalence of health conditions in brachycephalic breeds which often results in high‐cost surgical intervention (Fawcett et al. 2019). Lack of knowledge in this area may result in the owner being unprepared for these costs resulting in the dog not getting the treatment necessary thus impacting welfare.

Both dog owners and non‐dog owners had a statistically significant increase in their reported understanding of health issues after viewing the information sheet. This suggests the level of content provided was sufficient to improve the understanding of health issues amongst non‐brachycephalic breed owners. However, there was not a statistically significant change in reported understanding for owners of brachycephalic breeds. In contrast, the study by Kenny et al. (2022) found that their educational tool improved awareness of health‐related issues in 82.4% of owners of brachycephalic breeds. This difference may be due to the level of medical and scientific detail provided which primarily focused on BOAS in that study. This could suggest that a more advanced level of information may be needed to develop the knowledge of brachycephalic dog owners who rated themselves as significantly more knowledgeable prior to the intervention compared to those who did not own a dog or those who owned non‐brachycephalic breeds.

Prior to the intervention, there was no significant difference between non‐dog owners and dog owners in their reported level of support for continued breeding of brachycephalic breeds. Similarly, no significant difference was found between non‐dog owners and owners of brachycephalic breeds. However, owners of brachycephalic breeds had a statistically higher level of support for the continuation of breeding than dog owners. Therefore, despite owners of brachycephalic breeds having a greater awareness of health issues, this does not seem to dissuade them from supporting continued breeding (Steinert et al. 2019). This suggests that even though owners of brachycephalic breeds are aware of health difficulties, they feel the breed offers other benefits which outweigh the health concerns. There was a statistically significant increase in opposition to breeding for owners of non‐brachycephalic breeds following the intervention. However, non‐dog owners and owners of brachycephalic breeds showed no statistically significant change in support level for breeding after reading the information sheet. This was consistent with Kenny et al. (2022) where 70.7% of owners of brachycephalic breeds reported that the infographic on BOAS would not have changed their perception of the breed before getting their dog. This suggests that information alone may be insufficient to sway opinions of owners of brachycephalic breeds and people actively considering ownership of this breed category (Sandøe et al. 2017). Another reason for the lack of change in the opinions of owners of brachycephalic breeds could be the strength of their bond with their dog, as acknowledging their pet as unhealthy might elicit distress. Existing research has shown a strong bond forms between brachycephalic breeds and their owners (Packer et al. 2019). This could be a result of purposeful breeding for a cute look and favourable behavioural characteristics (Teng et al. 2016). However, another paradox arises here as developing the preferred breed characteristics to build bond also increases the health problems (Packer et al. 2019). As brachycephalic breeds remain popular, it has been suggested that cognitive dissonance amongst owners may result in them not accepting their dog suffers from severe health problems (Kenny et al. 2022). This is further supported by the finding of Packer et al. (2012) where 58% of owners of dogs affected by BOAS reported their dog did not have a breathing problem. Alternatives such as legislative changes may be required to protect these animals in light of the overwhelming scientific opinion that the dogs are suffering (Packer et al. 2017).

The findings of the present study suggest that while reading a minimalistic information sheet can change the attitudes of some groups, it is not sufficient to shift the attitudes of others. It is therefore important to consider additional and alternative ways to educate current and prospective dog owners so that welfare can be improved. However, a lack of research measuring the impact of different information intervention styles means it is difficult to prescribe the adequate level and style of information needed to change owners of brachycephalic breeds’ opinions, although Kenny et al. (2022) suggested greater detail was more effective. Bognár and Kubinyi (2023) suggested it may be necessary to highlight the significant role that dog owners play as consumers in shaping breed health. Owner purchasing choices directly influence breeding practices, and emphasising owner ability to demand healthier breeding standards could help mitigate the negative consequences of breed‐related health issues. The delivery method of the educational information must also be considered. Where prospective owners conduct pre‐purchase research, this is often through social media groups of brachycephalic breed owners, offering a unique opportunity to spread awareness of health issues (Phillips et al. 2024). However, the effect might not be as great as anticipated as Packer et al. (2017) found many existing owners would recommend their breed to a friend or family member regardless of brachycephalic health characteristics. It has also been suggested that a more fundamental cultural change is needed to reduce demand for these animals. Owners of brachycephalic breeds may overlook or be unaware that their choices encourage breeders to continue their current practices (Packer et al. 2020). Without a change in consumer behaviour, breeders will have no incentive to change breeding practices towards more healthy individuals.

One key limitation of the present study was the reliance on convenience and snowball sampling through social media platforms, which reduces the ability to generalise and interpret the findings. Participants recruited via these methods are likely to form a self‐selecting group, which could skew the sample towards individuals with a specific interest in or experience with brachycephalic breeds. Furthermore, as the study relies on self‐reported data, participants may have provided socially desirable answers especially if they perceive certain responses as more acceptable (Bohner and Dickel 2011). Participants may have been reluctant to report changes in their opinions regarding their own dogs due to psychological discomfort, which could lead to underreporting in areas which may reflect negatively on their decisions for the health of their pets.

While the educational intervention addressed multiple health issues and the background of brachycephalic breeds, it was limited to a simple design. Therefore, this format may not be sufficient to change deeply held beliefs or perceptions. Additionally, given the complexity associated with many of these health issues, it may require more comprehensive educational approaches to fully cover and effectively alter public opinion and behaviour. Finally, the population of the present study was not representative of dog owners or owners of brachycephalic breeds due to the small sample, so data may not be generalisable. Furthermore, there were only a few individuals in the sample population who did not own a dog meaning the statistical analysis may have lacked power. Demographic variables such as gender and socioeconomic status were not collected in this study, so it is possible that these may have been confounding factors.

In conclusion, a simple educational intervention has the ability to improve awareness of health issues in brachycephalic breeds and change attitudes to breeding amongst some groups but less so amongst others. Owners of brachycephalic breeds had the highest self‐reported awareness of the health issues of these breeds but were not significantly impacted by an educational intervention and maintained their support for continued breeding of these dogs.

## Author Contributions

Gareth Smith and Alison Wills both contributed to study design. Gareth Smith administered the survey and analysed the data. Gareth Smith and Alison Wills both contributed to the manuscript creation.

## Ethics Statement

Ethics approval for this study was granted by Hartpury University Ethics Committee (ETHICS2023‐284‐LR).

## Conflicts of Interest

The authors declare no conflicts of interest.

## Data Availability

The data that support the findings of this study are available on reasonable request by contacting the corresponding author.
